# RPA Plasmons in Graphene Nanoribbons: Influence of a VO_2_ Substrate

**DOI:** 10.3390/nano12162861

**Published:** 2022-08-19

**Authors:** Mousa Bahrami, Panagiotis Vasilopoulos

**Affiliations:** 1 Bita Quantum AI Inc., 2021 Av. Atwater, Montréal, QC H3H 2P2, Canada; 2 Department of Physics, Concordia University, 7141 Sherbrooke Ouest, Montreal, QC H4B 1R6, Canada

**Keywords:** plasmon, graphene nanoribbon, random-phase approximation, quantum wire, VO_2_, phase-change materials, normalized Fermi velocity

## Abstract

We study the effect of the phase-change material VO${}_{2}$ on plasmons in metallic arm-chair graphene nanoribbons (AGNRs) within the random-phase approximation (RPA) for intra- and inter-band transitions. We assess the influence of temperature as a knob for the transition from the insulating to the metallic phase of VO${}_{2}$ on localized and propagating plasmon modes. We show that AGNRs support localized and propagating plasmon modes and contrast them in the presence and absence of VO${}_{2}$ for intra-band (SB) transitions while neglecting the influence of a substrate-induced band gap. The presence of this gap results in propagating plasmon modes in two-band (TB) transitions. In addition, there is a critical band gap below and above which propagating modes have a linear negative or positive velocity. Increasing the band gap shifts the propagating and localized modes to higher frequencies. In addition, we show how the normalized Fermi velocity increases plasmon modes frequency.

## 1. Introduction

All modern electronic devices are based on semiconductors for which miniaturization means increased speed for processing and transferring information [1,2]. Advances in many areas of science and technology are intertwined with transferring and processing information. According to Moore’s law, the density of electronic elements in a circuit is doubled every two years. However, fundamental laws of quantum mechanics, thermodynamics, and electrodynamics prevent any arbitrary size reduction [3,4,5]. Fiber optics is an alternative to traditional electric circuits. By replacing electrons with light as the information carrier, one is able to transfer information about a thousand times faster than electronic devices [6]. This suggests that it may be possible to synergize these two technologies. However, due to the diffraction limit, which does not allow the localization of an electromagnetic field in an area smaller than its own wavelength, their integration is not possible [7,8].

Plasmonic waves, including plasmons, surface plasmon polaritons, as well as localized plasmons, enable us to manipulate, route and control light in the subwavelength area [7,9,10,11,12] though the large propagation loss of plasmonic waves limits their applications [13]. On the other hand, dielectric materials with low confinement compared to plasmonic systems suggest a hybrid system of these technologies [14]. The quality factor of an isolated hybrid structure, however, is low [15,16,17,18]. This disadvantage can be resolved by using a periodic array of hybrid structures, known as a metastructure. The periodicity in these metastructures must be smaller than the wavelength of the external stimuli including light, heat, electric field and so forth [19]. The unique and unprecedented properties of metastructures depend on the size, shape, composition, and environment of individual structures; by altering any of these factors one would be able to manipulate and control the outgoing radiation field as the response of the metastructure [19,20,21].

It is worth pointing out that these knobs and parameters are static and do not allow the manipulation of system responses in real time. In other words, metastructures are not tunable dynamically. Dynamically tunable metastructures are achievable by exploiting phase-change materials (PCMs) [22]. Due to their transition phases, from insulator to metal, in response to external perturbations, PCMs enable us to control and manipulate the phase, amplitude, and polarization of the outgoing electromagnetic field of the metastructure. Among PCMs, VO${}_{2}$, due to its ultrafast phase change and the large discontinuity in its optical properties at ambient pressure, has garnered tremendous interest during the last decade [23]. The typical critical temperature (T${}_{c}$) of VO${}_{2}$, is 340 K. Above this temperature it is in its metallic phase [24] that varies based on the fabrication of VO${}_{2}$ samples [25]. In this phase VO${}_{2}$ has a rutile structure, known as R, with periodic chains of vanadium ions parallel to c-axis [26] Below T${}_{c}$, it is in its insulating (semiconducting) phase with an optical gap of the order of 0.6 eV [25]. In this phase, due to dimerized zigzag chains of vanadium ions, it has a monoclinic structure known as M${}_{1}$ [27]. In addition, VO${}_{2}$ under uniaxial pressure or in doped samples undergoes a transition from R phase to the monoclinic M${}_{12}$ and triclinic T phases [27,28]. After five decades, the nature of these transitions due to electron–electron (Mott) and electron–lattice (Peierls) interactions is still not fully understood [25,29]. There are several methods for trigging the phase-transition in VO${}_{2}$ including electrical [30,31], thermal [32,33,34,35], optical [36,37,38,39], etc.

**Figure 1:**
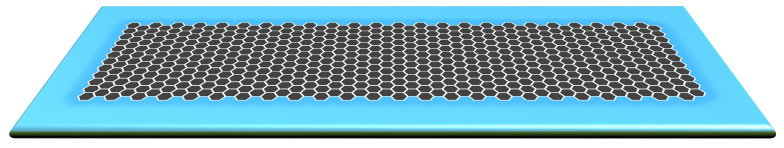
An AGNR on a VO${}_{2}$ substrate.

In this work we study plasmon modes in an AGNR on a VO${}_{2}$ substrate as sketched in Figure 1. The paper is organized as follows. In Section 2 we present the Lindhard polarization function and the RPA permittivity of AGNRs for SB and TB transitions. We also indicate briefly how to include the effect of a substrate on the plasmon modes and give the permittivity of VO${}_{2}$ obtained approximately in Reference [40]. Section 3 discusses the obtained results for localized and propagating plasmon modes in the presence and absence of VO${}_{2}$ for SB and TB transitions. In Section 4, we study the influence of a band gap, induced by a VO${}_{2}$ substrate, on the dielectric function and plasmon modes. The influence of renormalized Fermi velocity has been discussed in Section 5. A summary follows in Section 6.

## 2. Formalism

The main motivation to investigate plasmons in metallic AGNRs is that the conduction and valence bands of these systems, within the two-band approximation, are linear [41]. In general, to obtain the plasmon spectrum of a quantum system, one needs to find the roots of the real part of its permittivity [42]. It is worth pointing out that two types of plasmons exist; localized and propagating. The imaginary part of permittivity represents plasmon dissipation. Furthermore, the amount of dissipation for localized modes is much higher than for the propagating ones. Here, we limit our calculation to the RPA and we neglect the exchange–correlation effect which can be introduced by a local field factor, for example, in the Hubbard approximation [43]. The permittivity, also known as the dielectric function in the momentum and frequency domains within the RPA, is given by [42]:

(1) $$\epsilon^{RPA}\left(\vec{q},\omega \right)=1-V\left(\vec{q}\right)\chi \left(\vec{q},\omega \right),$$

where $V(\vec{q})$ and $\chi (\vec{q},\omega )$ are the matrix elements of the Coulomb potential and the Lindhard polarization function, respectively. The linear eigenvalues of metallic AGNRs support two sorts of transitions; intra-band and inter-band transitions, also known as single-band (SB) and two-band (TB) transitions, respectively. To distinguish between the plasmon modes for these transitions, we use the corresponding polarization functions $\chi^{SB}$ and $\chi^{TB}$ from Reference [44], namely:

(2) $$\begin{array}{c} \chi^{SB}\left(q^{\prime },\omega^{\prime }\right)=\frac{1}{2\pi \hbar v_{F}^{*}}\;\\ \times \left\{\begin{array}{ccc} \frac{2{q^{\prime }}^{2}}{{\omega^{\prime }}^{2}-{q^{\prime }}^{2}}+i\pi q^{\prime }\left[\delta \left(q^{\prime }+\omega^{\prime }\right)-\delta \left(q^{\prime }-\omega^{\prime }\right)\right]\; & \; & q\leq k_{F},\;\\ \frac{2q^{\prime }}{{\omega^{\prime }}^{2}-{q^{\prime }}^{2}}+\frac{1}{2}Ln\left[\frac{{\left(2+\omega^{\prime }\right)}^{2}-{q^{\prime }}^{2}}{{\omega^{\prime }}^{2}-{q^{\prime }}^{2}}\right]+i\pi \left[\delta \left(q^{\prime }+\omega^{\prime }\right)-\delta \left(-q^{\prime }+\omega^{\prime }\right)\right]\; & \; & q>k_{F}, \end{array}\right.\; \end{array}$$

(3) $$\begin{array}{c} \chi^{TB}\left(q^{\prime },\omega^{\prime }\right)=\frac{1}{2\pi \hbar v_{F}^{*}}\;\\ \times \left\{\begin{array}{ccc} \frac{2{q^{\prime }}^{2}}{{q^{\prime }}^{2}-{\omega^{\prime }}^{2}}+\frac{1}{2}Ln\left(\frac{9{q^{\prime }}^{2}-{\omega^{\prime }}^{2}}{{q^{\prime }}^{2}-{\omega^{\prime }}^{2}}\right)+i\pi q^{\prime }\left[\delta \left(q^{\prime }-\omega^{\prime }\right)-\delta \left(q^{\prime }+\omega^{\prime }\right)\right] & \; & q\leq k_{F},\;\\ \frac{2q^{\prime }}{{q^{\prime }}^{2}-{\omega^{\prime }}^{2}}+\frac{1}{2}Ln\left(\frac{{\left(2+q^{\prime }\right)}^{2}-{\omega^{\prime }}^{2}}{{q^{\prime }}^{2}-{\omega^{\prime }}^{2}}\right)+i\pi \left[\delta \left(q^{\prime }-\omega^{\prime }\right)-\delta \left(q^{\prime }+\omega^{\prime }\right)\right] & \; & q>k_{F}, \end{array}\right.\; \end{array}$$

where $q^{\prime }=q/k_{F}$, $\omega^{\prime }=\hbar \omega /E_{F}$, and $v_{F}^{*}$ are the dimensionless wave vector, frequency, and renormalized Fermi velocity respectively. One should notice that any substrate modifies the Fermi velocity of graphene ribbons, and it can be obtained through the THz-TDS measurement schemes [45]. It is worth pointing out that any substrate can introduce a gap in the energy spectrum of AGNRs similar to graphene on hBN or any TMDC material [46,47,48,49]. In general, this introduced gap will not alter the polarization function for SB transitions. In addition, one can take into account this effect for TB transitions by replacing $\omega^{\prime }$ with $\omega^{\prime }+\Delta^{\prime }$ with $\Delta^{\prime }\equiv \Delta /E_{F}$ being the gap energy. First, we consider $\Delta^{\prime }=0$ and in Section 4 take $\Delta^{\prime }\neq 0$.

**Figure 2:**
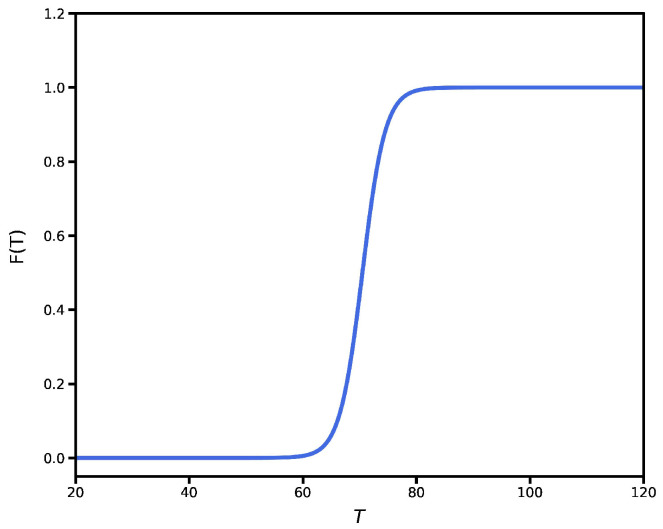
VO${}_{2}$ filling factor as a function of temperature.

The Coulomb matrix element in Equation (1) for both SB and TB transitions is given by [50]:

(4) $$\begin{array}{c} V\left(q\right)=\frac{2e^{2}}{\epsilon_{0}\epsilon_{b}}\int_{0}^{1}\int_{0}^{1}K_{0}(Wk_{F}q^{\prime }|y-y^{\prime }\left|\right)dydy^{\prime }, \end{array}$$

where *W* and $K_{0}$ are the ribbon width and the zeroth order modified Bessel function of the second kind, respectively. Note that in expression (4), the $\epsilon_{b}$ parameter is the averaged permittivity defined by $\epsilon_{b}=\left(1+\epsilon_{VO_{2}}\right)/2$ [51]. Since the temperature strongly affects this parameter, it can act as a knob to switch on and off the plasmon modes. One successful method in describing the temperature dependence of VO${}_{2}$ is the Maxwell-Garnet approximation explained in Reference [40]. In it the permittivity is:

(5) $$\epsilon_{MG}=\epsilon_{ins}\frac{\epsilon_{met}\left(2F+1\right)+\epsilon_{ins}\left(2-2F\right)}{\epsilon_{met}\left(1-F\right)+\epsilon_{ins}\left(2+F\right)},$$

where *F*, the filling factor, is the ratio between the metallic and insulating phases. It is given by [52]:

(6) $$F\left(T\right)=\frac{1}{1+e^{2\left(T_{0}-T\right)/T_{c}}},$$

with $T_{0}$ = 70.5 °C and $T_{c}$ = 4 °C. In Figure 2, we plot the filling factor as a function of the temperature *T*. As shown, F=0 for T<60 °C. In this range, VO${}_{2}$ is in the insulating phase while for T>80 °C it is in the metallic phase. Moreover, for 60<T<80 it is in a mixture of both phases.

In the following, we consider $v_{F}^{*}=v_{F}=10^{6}$ m/s for simplicity. The influence of the renormalized Fermi velocity will be discussed in Section 5.

## 3. Results and Discussion

**Figure 3:**
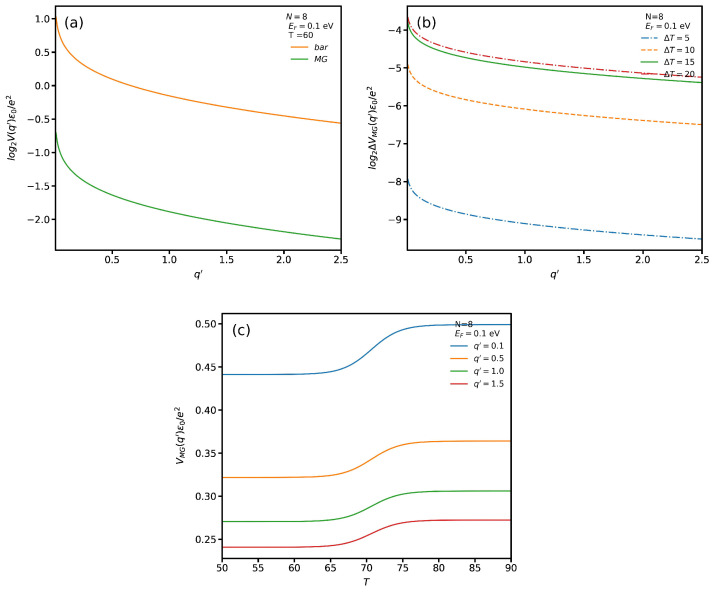
(**a**) Coulomb potential in AGNRs in the absence (bar) and presence (MG) of VO${}_{2}$; (**b**) $\Delta V(q^{\prime })$ for several ΔT; (**c**) $V_{MG}\left(q^{\prime }\right)$ as a function of temperature for several wave vectors.

In Figure 3a, we plot the Coulomb potential $V(q^{\prime })$ as a function of the dimensionless wave vector $q^{\prime }=q/k_{F}$ in the absence and presence of a VO${}_{2}$ substrate; the Fermi energy and ribbon width dimer numbers are $E_{F}=0.1$ eV and N=8, respectively. Here the average dielectric constant, $\epsilon_{b}$, is considered at T=60 °C in which the substrate is in insulating phase. Since the difference between these two cases is very small, we utilize the logarithmic scale. Here *bar* and *MG* indicate the presence and absence of VO${}_{2}$, respectively. As shown, the Coulomb potential in both cases shows the same trend, i.e., by increasing the wave vector its magnitude reduces rapidly. To investigate the impact of temperature on the $V(q^{\prime })$, in Figure 3b we plot the difference between $V(q^{\prime })$ at T=60 °C and that at several temperatures specified by ΔT in the figure. It is noticeable that by increasing the temperature, which is equivalent to increasing the fraction of the metallic, the magnitude of the Coulomb potential becomes larger. In Figure 3c we plot Coulomb potential as a function of the temperature for several values of the wave vector. We see that $V(q^{\prime })$ has a lower magnitude in the insulating phase than in the metallic one. In addition, increasing the wave vector leads to a decrease of the $V(q^{\prime })$ magnitude.

**Figure 4:**
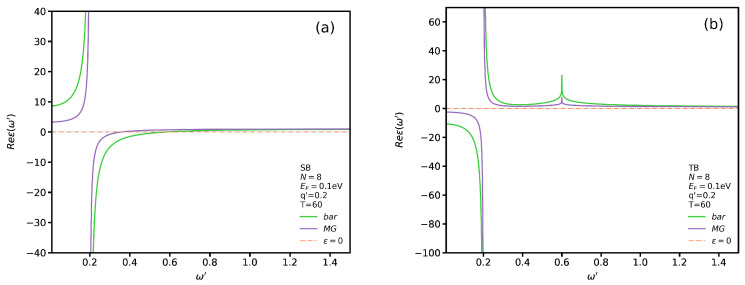
Real parts of $\epsilon^{RPA}$, at T=60 °C as functions of $\omega^{\prime }$ for SB transitions in (**a**) and TB transitions in (**b**).

In Figure 4a,b, we plot the real parts of Equation (1) for SB and TB transitions, respectively, as functions of the dimensionless frequency $\omega^{\prime }$, in the presence and absence of VO${}_{2}$, and for a typical wave vector, for example, $q^{\prime }=0.2$. In addition, we plot the orange line ϵ=0 to see where the zeros of $\epsilon^{RPA}$ occur.

We notice that, for SB transitions, the graphs intersect the orange line at two points which are related to localized and propagating plasmon modes in the presence and absence of VO${}_{2}$. However, for TB transitions there is only one intersection point due to localized plasmon modes. In addition, for SB transitions we see in Figure 4a that the graphs in the presence and absence of VO${}_{2}$ for low frequencies increase and decrease dramatically. In general, the magnitude of the AGNR permittivity in the absence of VO${}_{2}$ is higher. Moreover, the permittivity for TB transitions, compared to that for SB transitions, shows a reverse behavior. Indeed, for low frequency, the magnitude of the AGNR permittivity in the presence of VO${}_{2}$ is higher than in its absence.

**Figure 5:**
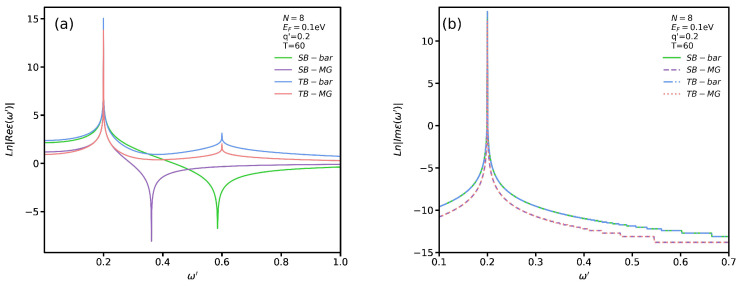
Real (**a**) and imaginary (**b**) parts of $\epsilon^{RPA}$ as functions of $\omega^{\prime }$ for both SB and TB transitions.

To better assess the effect of VO${}_{2}$’s presence on $\epsilon^{RPA}$, in Figure 5a,b we plot, respectively, its real and imaginary parts for SB and TB transitions with $q^{\prime }=0.2$ and T=60 °C. We employ the logarithmic scale to see more clearly the subtle difference between these transitions. It is noticeable that the permittivity diverges in all cases for $\omega^{\prime }=0.2$. If one looks at the imaginary part of the permittivity at this point in Figure 5b, one can observe that the magnitude has the highest value which indicates a maximum dissipation at this frequency. In other words, if a plasmon excitation mode occurs at this point, due to its high dissipation it would definitely be a localized one. We observe that the real part of the dielectric function for both SB and TB transitions diverges at two points which are pertinent to localized plasmon and single-body excitation modes. The difference between these modes can be distinguished by comparing the magnitude of $Im\epsilon^{RPA}$. For instance, the two points for TB transitions in the absence of VO${}_{2}$ occur at $\omega^{\prime }=0.2$ and $\omega^{\prime }=0.6$. If one looks at the magnitude of $Im\epsilon^{RPA}$ at these two frequencies, one can see that the magnitude at $\omega^{\prime }=0.2$ is much higher than that at $\omega^{\prime }=0.6$. Therefore, on can conclude that the first and second points are relevant to the localized plasmon and single-body excitation modes, respectively. In addition, we notice that the single-body excitation mode in the presence of VO${}_{2}$ occurs at a lower frequency for SB transitions. In other words, the substrate causes a significant shift in the permittivity spectrum. However, the divergence point for TB transitions does not shift. We notice that in Figure 5b the magnitude of $Im\epsilon^{RPA}$ vanishes except at a specific point. The interesting trend is the similarity between the behaviors of the graphs, in the presence and absence of VO${}_{2}$, for SB and TB transitions. In particular, the magnitude of the imaginary part of the permittivity has the same value in the absence and presence of VO${}_{2}$ regardless of the transition type.

**Figure 6:**
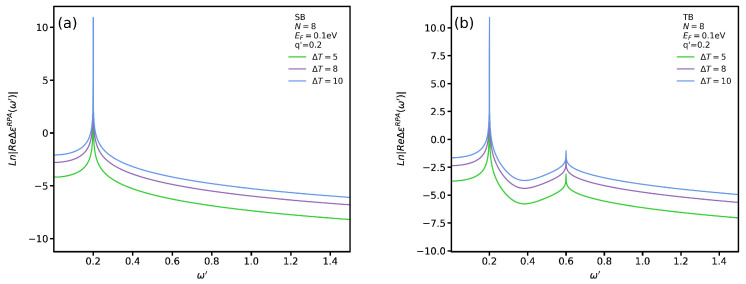
Real part of $\Delta \epsilon^{RPA}$ as function of $\omega^{\prime }$ for (**a**) SB and (**b**) TB transitions.

To assess the influence of the temperature *T* on the permittivity we plot $\Delta \epsilon_{MG}\equiv \epsilon^{RPA}\left(T\right)-\epsilon^{RPA}\left(60\right)$ in Figure 6a,b for SB and TB transitions, respectively. In general, we see that increasing *T* causes a transition from the insulating to the metallic phase in VO${}_{2}$ and the magnitude of the permittivity increases for both transitions. In addition, we observe that the divergence points do not shift by increasing the temperature.

**Figure 7:**
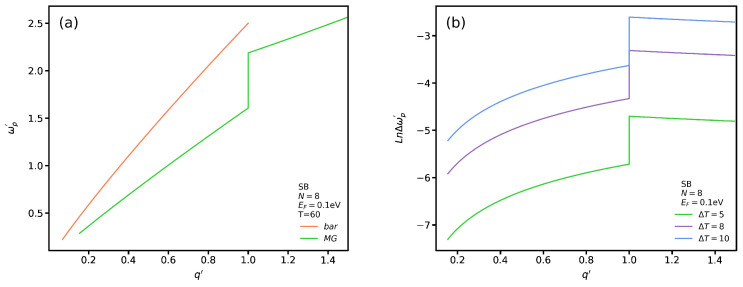
(**a**) Propagating plasmon for SB transition spectrum in the absence and presence of VO${}_{2}$. (**b**) $\omega_{p}^{\prime }$ for several ΔT.

In Figure 7a we plot the propagating plasmon modes for SB transition. The labels *bar* and *MG* distinguish between including and excluding the VO${}_{2}$ substrate. Note that there is no propagating plasmon modes for TB transition (see Figure 7b). The principal reason for using these labels is to show the impact of VO${}_{2}$ on the Coulomb matrix elements given by Equation (4). As shown in Figure 7a, we see that the slope of the *SB-bar* transition, in the absence of a VO${}_{2}$ substrate, is higher than that in its presence, *SB-MG*. In other words, the excitation energy of the propagating plasmon is higher than that of the *SB-MG* one for the same wave vector. While the plasmon mode dispersion of *SB-bar* is limited to wave vectors smaller than the Fermi wave vector, the *SB-MG* plasmon mode dispersion exists for all wave vectors. In addition, the *SB-MG* plasmon mode energy has an unexpected upward jump at the Fermi wave vector $q^{\prime }=1$. It is also noticeable that *SB-MG* plasmons are absent for $q^{\prime }\leq 0.18$ though *SB-bar* plasmons exist in this range of wave vectors.

**Figure 8:**
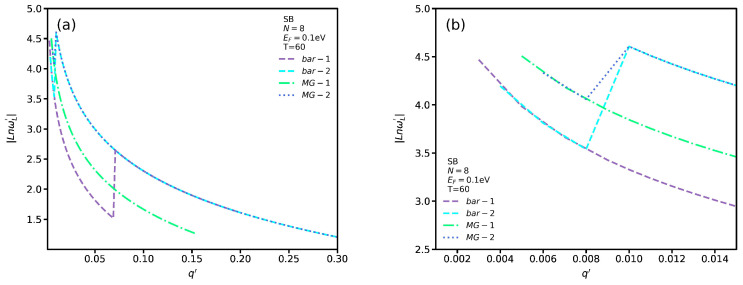
(**a**) Localized plasmon and single-body excitation dispersion for SB transitions in the absence and presence of VO${}_{2}$; (**b**) As in (a) for $q^{\prime }\leq 0.15$.

To show the difference between propagating SB plasmon modes for different temperatures, we set $\Delta \omega_{p}^{\prime }$ as the difference between the plasmon frequency at temperatures *T* and T=60 °C in Figure 7b. In addition, we use the logarithmic scale to plot the $\Delta \omega_{p}^{\prime }$ dispersion. Figure 7b shows a similar trend for $\Delta \omega_{p}^{\prime }$ spectrum in which increasing the wave vector magnitude leads to an increase of $\Delta \omega_{p}^{\prime }$ for $q^{\prime }\leq 1$ whereas for $q^{\prime }\geq 1$ there are no significant changes. Furthermore, increasing ΔT results in larger $\Delta \omega_{p}^{\prime }$ values. Additionally, the slope of the dispersion decreases with the plasmon wave vector for $q^{\prime }\geq 1$. In Figure 8a, we plot the localized plasmon and single-body excitation dispersion in the presence and absence of VO${}_{2}$ for SB transitions. In Figure 8b, we plot the SB spectrum in the long wavelength limit to see their behavior more clearly. The labels *bar-1* and *bar-2* indicate the localized plasmon and single-body excitation branches for SB in the absence of a VO${}_{2}$ substrate and *MG-1*, *MG-2* those in its presence. As seen, the *bar-1* branch decreases rapidly by increasing $q^{\prime }$. However, near $q^{\prime }=0.07$ there is a jump in which the energy of the localized plasmon increases unexpectedly. Then, the previous trend in *bar-1* continues but the group velocity becomes lower. In Figure 8b, there is a degeneracy range between the *bar-1* and *bar-2* branches in which their spectra have the same frequency and momentum. Returning to Figure 8a, there is another degeneracy range which begins exactly after the jumping point in the *bar-1* branch. Note that the *bar-1* branch extends only for a limited range in which the maximum wave vector is $q^{\prime }=0.2$. For the *bar-2* branch, we observe a similar pattern but the jumping point in *bar-1* occurs at higher $q^{\prime }$. In the presence of VO${}_{2}$, the first *MG-1* branch is similar to the *bar-1* one in which the localized plasmon frequency is reduced by increasing the wave vector. Nevertheless, the *MG-1* frequencies are higher than those of the *bar-1* branch. In addition, we note that, in this case, there exists only one degeneracy range, whereas in the absence of VO${}_{2}$ there are two ranges. As shown in Figure 8b, the *MG-2* plasmon spectrum coincides with that of the *bar-2* one except in the long wavelength limit. For TB transitions, as we mentioned in Figure 4, there are no propagating plasmon modes and only localized plasmon modes exist whose slope is equal to one. Hence, we have not included them here.

## 4. Band Gap Effect on Polarization and Plasmon Modes

Some studies show that substrates and nanostructuring (by e.g., creating nanoribbons) induce a band gap between the conduction and valence bands in graphene [46,47,48,49,53,54,55,56,57]. To account for the influence of this gap in the AGNR’s polarization function, it suffices to change ω to ω+Δ in the denominator of Equation (19) in Reference [44]. While the results are the same as in Equation (2) for SB transitions, for TB transitions we obtain:

(7) $$\begin{array}{c} \chi^{TB}\left(q^{\prime },\omega^{\prime }\right)=\frac{1}{2\pi \hbar v_{F}}\;\\ \times \left\{\begin{array}{ccc} \frac{2q^{\prime }\left(q^{\prime }+\Delta^{\prime }\right)}{{\left(q^{\prime }+\Delta^{\prime }\right)}^{2}-{\omega^{\prime }}^{2}}+\frac{1}{2}Ln\left(\frac{{\left(3q^{\prime }+\Delta^{\prime }\right)}^{2}-{\omega^{\prime }}^{2}}{{\left(q^{\prime }+\Delta^{\prime }\right)}^{2}-{\omega^{\prime }}^{2}}\right)+i\pi q^{\prime }\left[\delta \left(q^{\prime }+\Delta^{\prime }-\omega^{\prime }\right)-\delta \left(q^{\prime }+\Delta^{\prime }+\omega^{\prime }\right)\right] & \; & q\leq k_{F},\;\\ \frac{2(q^{\prime }+\Delta^{\prime })}{{\left(q^{\prime }+\Delta^{\prime }\right)}^{2}-{\omega^{\prime }}^{2}}+\frac{1}{2}Ln\left(\frac{{\left(2+\Delta^{\prime }+q^{\prime }\right)}^{2}-{\omega^{\prime }}^{2}}{{\left(q^{\prime }+\Delta^{\prime }\right)}^{2}-{\omega^{\prime }}^{2}}\right)+i\pi \left[\delta \left(q^{\prime }+\Delta^{\prime }-\omega^{\prime }\right)-\delta \left(q^{\prime }+\Delta^{\prime }+\omega^{\prime }\right)\right] & \; & q>k_{F}, \end{array}\right.\; \end{array}$$

**Figure 9:**
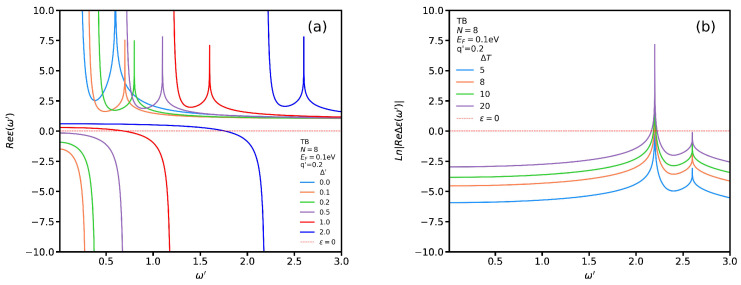
(**a**) Real part of $\epsilon^{RPA}$ as functions of $\omega^{\prime }$ for TB transitions for several $\Delta^{\prime }$ at T=60 °C. (**b**) $\Delta \epsilon^{RPA}$ for several temperature for $\Delta^{\prime }=2.0$.

Hence, we need to focus on the influence of the substrate on the TB permittivity and plasmon modes hereafter. In Figure 9a we show the real part of the TB dielectric function for several $\Delta^{\prime }$ at T=60 °C. As seen, all graphs show the same trend. In particular, by increasing the gap, the localized plasmon modes occur at higher frequencies. In addition, we find out that there is a critical $\Delta_{c}^{\prime }$ below which no propagating plasmon modes exist while there are both localized plasmon and single-body excitation modes. For instance, we observe that for $\Delta^{\prime }<1$ at $q^{\prime }=0.2$ there are no propagating plasmon modes. In addition, the frequency of the propagating plasmon modes becomes higher by increasing $\Delta^{\prime }$. Moreover, we see that localized plasmon and single-body excitation modes shift to higher frequencies by increasing the band gap. We plot $\Delta \epsilon^{RPA}$ for several temperatures in Figure 9b for $\Delta^{\prime }=2.0$ at $q^{\prime }=0.2$. While one can observe the same trend as Figure 6b, one notices that the divergence points shift to higher frequencies.

**Figure 10:**
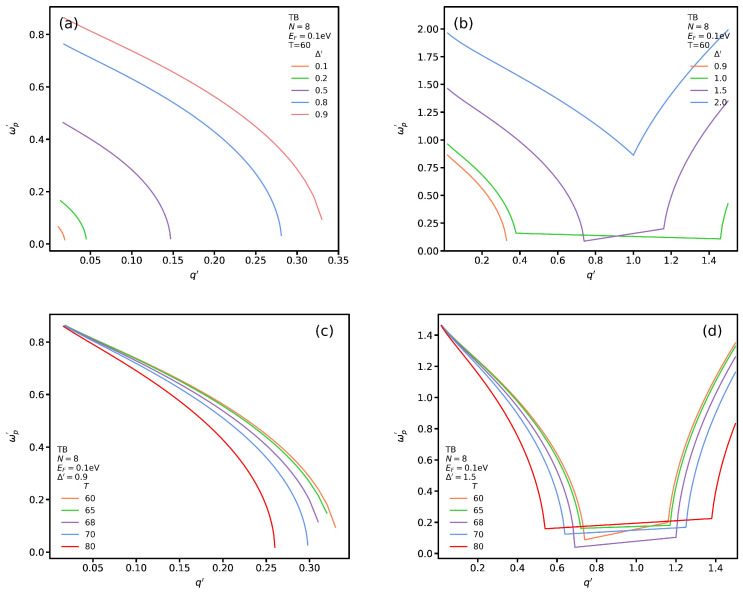
(**a**,**b**) propagating plasmon modes for different value of $\Delta^{\prime }\leq 1$ and $\Delta^{\prime }>1$ at T=60 °C for TB transition in the presence of VO${}_{2}$. (**c**,**d**) plasmon spectrum for several temperature for $\Delta^{\prime }=0.9$ and $\Delta^{\prime }=1.5$.

We plot the propagating plasmon modes for different values of $\Delta^{\prime }$ at T=60 °C for TB transitions in Figure 10a,b when $\Delta^{\prime }\leq 1$ and $\Delta^{\prime }>1$, respectively. Figure 10a shows that the propagating plasmon spectrum increases for higher values of $\Delta^{\prime }$. In particular, in the long-wavelength limit, the plasmon energy increases by $\Delta^{\prime }$. In addition, the group velocity of the plasmon modes is negative. As seen in Figure 10b, a parabolic trend of plasmon modes of $\Delta^{\prime }=1$ turns to a linear one with a negative slope at $q^{\prime }≃0.4$ and then, at $q^{\prime }≃1.45$, it shows again a parabolic behavior with positive group velocity. One can note that, by increasing the $\Delta^{\prime }$, the range of the linear trend becomes shorter and its group velocity changes from negative to positive. In addition, the linear trend vanishes. In this case the plasmon spectrum shows a negative velocity at the beginning but by increasing $q^{\prime }$ at a certain point it changes its behavior and manifests a positive group velocity. We plot the propagating plasmon dispersion, with $\Delta^{\prime }=0.9$, for several temperatures to asses how shifting from insulating to metallic phase of VO${}_{2}$ modifies the propagating plasmon spectrum in Figure 10c. One notices that all the graphs have the same trend, i.e., the propagating plasmon frequency decreases and by increasing its wave vector. In addition, the propagating plasmon spectrum’s range is reduced by increasing the temperature. In other words, increasing the temperature shifts the plasmon energy to lower values. Figure 10d depicts, as we explained in Figure 10c, when the energy gap is much larger than the Fermi energy ($\Delta^{\prime }=1.5$). One can observe that, for all temperatures, there is a general trend, which we mentioned in Figure 10b. However, one sees that the linear part of the plasmon spectrum increases for higher temperatures. In addition, the slope of this linear part of dispersion is higher for lower temperatures.

**Figure 11:**
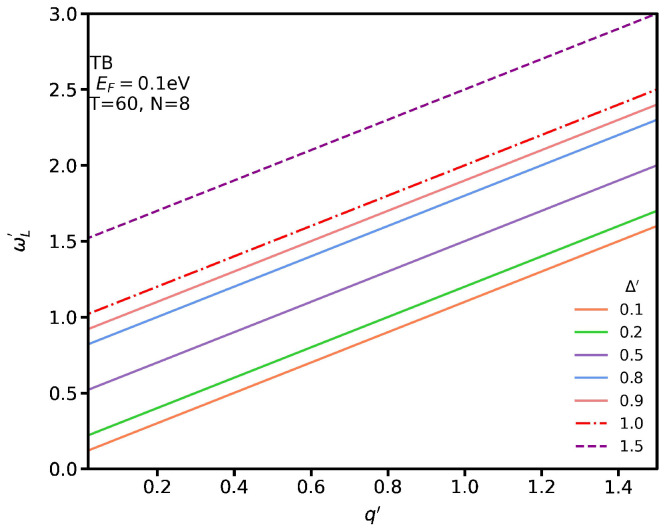
Localized plasmon dispersion for several $\Delta^{\prime }$ at T=60 °C.

Figure 11 shows the localized plasmon spectrum for several $\Delta^{\prime }$ at T=60 °C. One can observe that all graphs have the same slope. In addition, we see the frequency of the localized plasmon for $q^{\prime }$ becomes larger by increasing the energy gap. Here, we have not included the single-body excitation spectrum since the differences in temperature and $\Delta^{\prime }$ have a negligible effect on it.

## 5. Renormalized Fermi Velocity on Plasmon Modes

**Figure 12:**
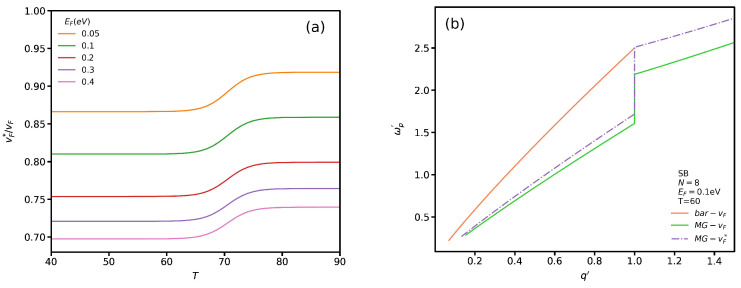
(**a**) Renormalized Fermi velocity as functions of temperature for several $E_{F}$; (**b**) Comparing SB plasmon spectrum for $v_{F}$ and $v_{F}^{*}$.

Indeed, the renormalized Fermi velocity of AGNRs in the presence of VO${}_{2}$ in both insulating and metallic phases can be evaluated by

(8) $$\begin{array}{c} \frac{v_{F}^{*}}{v_{F}}=1+\frac{\alpha }{\left(4+2\pi \alpha \right)}Ln\left(\frac{\Lambda \hbar v_{F}}{E_{F}}\right), \end{array}$$

where Λ=1.75Å${}^{-1}$ and $\alpha =2.2/\epsilon_{b}$ [45,58]. Here, we limit our study as an example only to the SB case to see the influence of $v_{F}^{*}$ on the plasmon spectrum. Figure 12a shows $v_{F}^{*}/v_{F}$ as a function of temperature for several Fermi energies. One can notice a general trend in which the normalized Fermi velocity has a lower value in the insulating phase than in the metallic phase. In addition, the normalized Fermi velocity decreases by increasing the Fermi energy. In Figure 12b, similar to Figure 7a, the labels *bar-$v_{F}$* and *MG-$v_{F}$* indicate the presence and absence of VO${}_{2}$ when $v_{F}^{*}=v_{F}$. The label MG-$v_{F}^{*}$ indicates the presence of VO${}_{2}$ when $v_{F}^{*}$ is evaluated by Equation (8). Although the MG-$v_{F}^{*}$ plasmon spectrum behavior is similar to that of *MG-$v_{F}$*, the latter plasmon energy is lower than the former. In particular, the difference between plasmon energy of *MG-$v_{F}$* and MG-$v_{F}^{*}$ increases by $q^{\prime }$ for $q^{\prime }\leq 1$. While this difference is constant for $q^{\prime }>1$.

## 6. Summary

We studied the effects of VO${}_{2}$ as a PCM on plasmons in metallic AGNRs within the RPA for both SB and TB transitions. We observed that metallic AGNRs support two types of plasmon modes: localized and propagating as well as single-body excitation modes for SB transitions while no propagating plasmon modes exist for TB transitions, provided the influence of a substrate-induced band gap is negligible ($\Delta^{\prime }≃0$). The group velocity of the localized plasmon modes and single-body excitations decreases with the plasmon wave vector whereas that of the propagating plasmons increases with it. We further showed how the transition from the insulating to the metallic phase in VO${}_{2}$ modifies the plasmon dispersion. For SB transitions, we noticed that the presence of VO${}_{2}$ results in propagating plasmons for a range of wave vectors which do not exist in the absence of VO${}_{2}$. In particular, a jumping point in the spectrum of the propagating plasmons exists at which the frequency increases unexpectedly.

We found that the presence of VO${}_{2}$ leads to localized plasmon modes with a higher frequency for SB transitions. In addition, the wave vector range becomes smaller and the jumping point disappears. We further notice that the single-body excitation spectrum does not change except in the long-wavelength limit. Further, we showed that the jumping point of the single-body excitations occurs at lower wave vectors in the absence of VO${}_{2}$ and a degeneracy exists between the localized plasmon and single-body excitation beyond the jumping point of the localized mode. However, in the presence of VO${}_{2}$, only one jumping point exists in the long-wavelength limit for single-body excitations. We showed that taking into account a substrate-induced band gap leads to a modification of the Lindhard polarization function which in turn leads to propagating plasmon modes for TB transitions. Increasing $\Delta^{\prime }$ shifts the frequencies of the propagating and localized plasmons as well as the single-body excitations to higher values. In addition, we noticed that a critical energy band gap, $\Delta_{c}^{\prime }$, exists below which the group velocity of the propagating plasmons is negative. Furthermore, we observed that the parabolic behavior of the propagating plasmon modes with negative group velocity becomes linear by increasing the value of $\Delta^{\prime }$ beyond $\Delta_{c}^{\prime }$. By further increasing $\Delta^{\prime }$, the spectrum acquires a positive group velocity. The range of the propagating plasmon spectrum increases for higher values of $\Delta^{\prime }$. Finally, increasing the temperature results in an extension of the linear part of the propagating-plasmon dispersion.

We found that the normalized Fermi velocity, $v_{F}^{*}$, has a higher value in the metallic phase than in the insulating phase. In addition, $v_{F}^{*}$ value increases by decreasing the Fermi energy. We showed that reducing the value of $v_{F}^{*}$ leads to plasmon modes with a higher frequency in the SB case.

The obtained results could be employed in plasmonic quantum processors in which modifying the temperature as a knob plays a critical role in altering qubits states.

## Data Availability

Not applicable.
